# SARS-CoV-2 Exacerbates Beta-Amyloid Neurotoxicity, Inflammation and Oxidative Stress in Alzheimer’s Disease Patients

**DOI:** 10.3390/ijms222413603

**Published:** 2021-12-19

**Authors:** Luigi Chiricosta, Agnese Gugliandolo, Emanuela Mazzon

**Affiliations:** IRCCS Centro Neurolesi “Bonino-Pulejo”, Via Provinciale Palermo, Contrada Casazza, 98124 Messina, Italy; luigi.chiricosta@irccsme.it (L.C.); agnese.gugliandolo@irccsme.it (A.G.)

**Keywords:** Alzheimer’s disease, SARS-CoV-2, next-generation sequencing, transcriptome, interactome, beta-amyloid, neurotoxicity, immune response, oxidative stress

## Abstract

Severe acute respiratory syndrome coronavirus 2 (SARS-CoV-2) triggered the pandemic Coronavirus Disease 19 (COVID-19), causing millions of deaths. The elderly and those already living with comorbidity are likely to die after SARS-CoV-2 infection. People suffering from Alzheimer’s disease (AD) have a higher risk of becoming infected, because they cannot easily follow health roles. Additionally, those suffering from dementia have a 40% higher risk of dying from COVID-19. Herein, we collected from Gene Expression Omnibus repository the brain samples of AD patients who died of COVID-19 (AD+COVID-19), AD without COVID-19 (AD), COVID-19 without AD (COVID-19) and control individuals. We inspected the transcriptomic and interactomic profiles by comparing the COVID-19 cohort against the control cohort and the AD cohort against the AD+COVID-19 cohort. SARS-CoV-2 in patients without AD mainly activated processes related to immune response and cell cycle. Conversely, 21 key nodes in the interactome are deregulated in AD. Interestingly, some of them are linked to beta-amyloid production and clearance. Thus, we inspected their role, along with their interactors, using the gene ontologies of the biological process that reveals their contribution in brain organization, immune response, oxidative stress and viral replication. We conclude that SARS-CoV-2 worsens the AD condition by increasing neurotoxicity, due to higher levels of beta-amyloid, inflammation and oxidative stress.

## 1. Introduction

The pandemic from severe acute respiratory syndrome coronavirus 2 (SARS-CoV-2) blew up in 2019, with more than 260 million infections and more than 5 million deaths around the world, according to the World Health Organization (WHO) [1]. SARS-CoV-2 is a virus of the betacoronavirus genus that triggers the Coronavirus Disease 19 (COVID-19) disease. COVID-19 consists of a respiratory disease that mainly causes fatigue, fever, dry cough and, in the worst cases, acute respiratory distress syndrome. Most of the people infected by this virus have an asymptomatic or paucisyntomatic course of the disease and, eventually, become healthy. In spite of this, 2% of people suffering from COVID-19 develop a severe disease that is associated with an uncontrolled inflammatory response and low oxygen levels in the blood that lead, in the worst cases, to respiratory failure, multi-organ impairment and, eventually, death [2]. The 35% of people that suffered from severe COVID-19 and become negative to SARS-CoV-2 still show, after two or more weeks, anosmia, headache, fatigue or joint pain linked to the so-called “long COVID-19”. The symptoms can vary, and they are related to brain, lungs, stomach, intestine, heart and more others tissue [3]. On the other hand, most of the people who died of COVID-19 were elderly people with one or more comorbidities that worsen the clinical condition. Among them, people suffering from cerebrovascular, cardiovascular disease, hypertension, obesity or diabetes have a bigger risk to develop a severe disease, due to their preclinical status [4].

Among neurological patients, people suffering from dementia are more than 55 million and have even a greater risk to be infected by SARS-CoV-2. WHO statistics associate it with Alzheimer’s disease (AD), the most common type of dementia that contributes to more than 60% of cases. One fact is that the AD rate increases with the age, but it is not limited to this [5]. AD is a chronic neurodegenerative disease that affects more than 46 million people in the world population and for which no cure is known. The causes of the disease are not clear, but beta-amyloid plaques and neurofibrillary tangles accumulation are clearly associated with the severity of the disease. AD implicates an alteration in memory and learning, as well as changes in normal behavior and in patient cognition [6]. Thus, the debilitating condition in which people with AD live hinders their ability to follow the health roles. Curiously, AD patients rarely show typical symptoms of the disease, such as cough or fever. Rather, diarrhea or drowsiness and, eventually, delirium triggered by hypoxia are observed. Different studies have focused on AD patients infected by the SARS-CoV-2, but, to date, it is not clear how the virus can modify the pre-existing clinical course of the disease [7]. However, it could be possible that COVID-19 triggers or accelerates neurological disorders, such as AD. COVID-19 and AD share several biochemical processes. Similar to AD, SARS-CoV-2 can alter the homeostasis of the blood–brain barrier, induce hypoxia and trigger neuroinflammation. It is notable that the angiotensin-converting enzyme 2 (ACE2) receptor is used by the virus as the entry point for human cells, and in AD patients, it seems to correlate with oxidative stress levels [8,9]. Since anosmia is one of the characteristic symptoms of the COVID-19, the olfactory epithelium, close to the frontal cortex, could represent one possible route for SARS-CoV-2 to enter into the brain [10]. In line with this, the frontal cortex is the brain area involved in working memory, planning and reasoning that becomes damaged in advanced AD [11]. For this reason, this brain area should highlight the highest link between SARS-CoV-2 and AD patients. Nevertheless, very few data are focused on elderly patients, and most of them are focused on patients without dementia comorbidity. Remarkably, it seems that people suffering from dementia showed a mortality-risk increase of 40% when compared to individuals without dementia; thus, the older age of these patients cannot fully explain the reason [12].

To inspect what the effects are that SARS-CoV-2 can trigger in patients with AD, in this work, we retrieved four cohorts of patients from the Gene Expression Omnibus [13] (GEO) repository. The first cohort is made of individuals who died of AD, whereas the second cohort is composed of people with AD who died of COVID-19. Additionally, a third cohort of control people and a fourth cohort of people without dementia who died of COVID-19 were retrieved. Then, through a next-generation sequencing analysis and an in silico study, we inspected the changes in the transcriptomic profile and in the interactomic network that are triggered in the frontal cortex area of the brain of the four cohorts of patients.

## 2. Results

The analysis performed on the genes of control cohort against the COVID-19 cohort highlighted 1644 differentially expressed genes (DEGs), among which 1384 are upregulated and 260 are downregulated. DEGs were uploaded on STRING, and all the nodes with degree 0 were removed. After setting the network with the highest level of confidence (0.900), along with “Experiments” and “Database” as sources, 1564 proteins remained. On the other hand, our comparison of the AD+COVID-19 cohort against the AD cohort revealed 904 upregulated and 843 downregulated DEGs, equaling 1747 overall DEGs. As with the previous analysis, STRING received in input DEGs and node with 0 degree were excluded. Confidence level and experimental sources kept 1689 proteins. The two comparisons of the four cohorts were then separately exported on Cytoscape, where the network analysis was performed. To inspect the nodes, we focused our attention on the betweenness centrality parameter that gives information about the centrality of a node in the graph. Specifically, the betweenness centrality of a given node is obtained through the amount of shortest paths that go through that node. Among the highlighted sub-networks, we focused our attention on DEGs in the subnetwork with the highest diameter. The main network coming from the control cohort against the COVID-19 cohort has a diameter of 18 and 157 proteins connected. We normalized the values of the betweenness centrality through z-score normalization, and we kept the node outer the 95% of the distribution (z-score > 1.96). Table 1 shows the 7 DEGs that we identified as central in the network. 

The comparison of the AD cohort against the AD+COVID-19 cohort revealed a main network diameter of 13 that connects 516 proteins. After z-score normalization of the betweenness centrality parameter, the 21 DEGs in Table 2 are in the upper 5% of the distribution.

**Figure 1:**
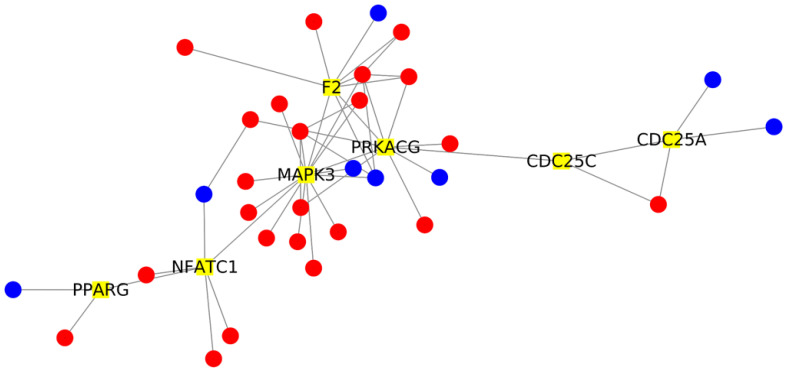
Network analysis of DEGs interacting with the 7 DEGs with the highest betweenness centrality. The network is composed of 38 nodes. The 7 key DEGs with the highest betweenness centrality are highlighted in yellow. The 31 interactor DEGs identified in the network are depicted in red if they are more expressed in the AD+COVID than AD cohort; otherwise, they appear in blue.

The key role of these highlighted genes in the network is due to their connectivity with other DEGs of the network. For this reason, we extrapolated from the network all the DEGs that directly interact with the DEGs in Table 1 that come from the control against COVID-19 comparison. We identified a total of 38 DEGs that were highlighted on STRING, using the same parameters; we then elaborated the final network with Cytoscape, and it is plotted in Figure 1.

**Figure 2:**
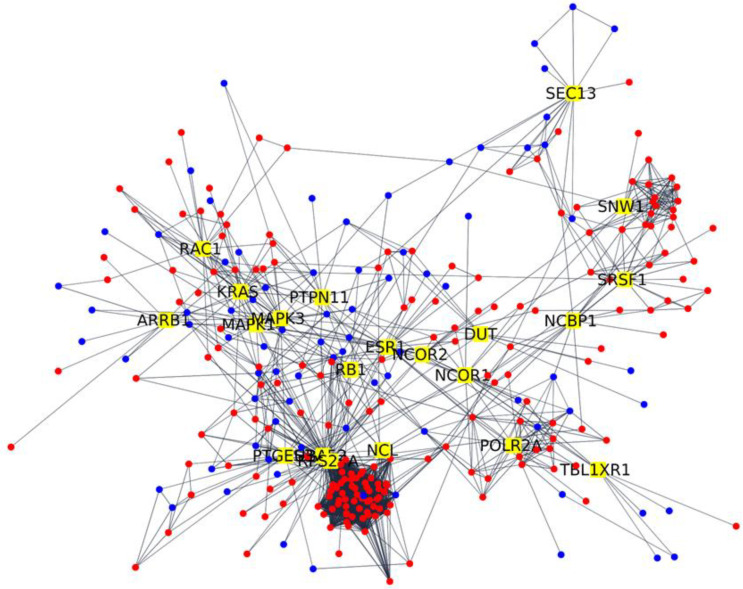
Network analysis of DEGs interacting with the 21 DEGs with the highest betweenness centrality. The network is composed of 265 nodes. The 21 key DEGs with the highest betweenness centrality are highlighted in yellow. The 244 interactor DEGs identified in the network are depicted in red if they are more expressed in the AD+COVID than AD cohort; otherwise, they appear in blue.

We made the same things for DEGs in Table 2 of the AD against AD+COVID-19 comparison, and the 265 DEGs are represented in Figure 2.

**Figure 3:**
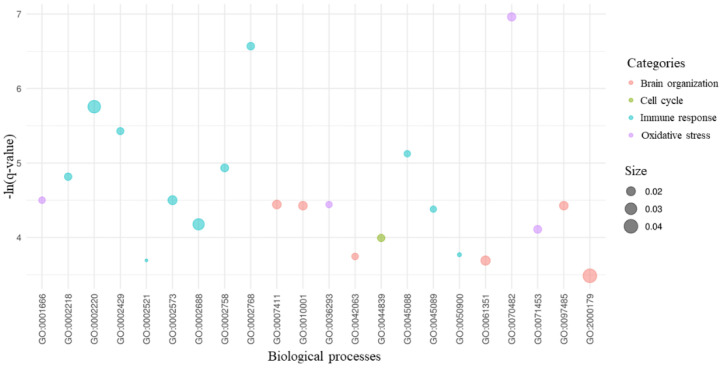
Biological processes of the key DEGs. For the 7 DEGs with the highest betweenness centrality and their 31 interactors, we provided the gene ontology enrichment of their biological processes. Among all the statistically significant processes, we kept the 22 processes related to brain organization, cell cycle, immune response and oxidative stress, as shown in the legend. On the *x*-axis are the gene ontology IDs of all the 45 processes. For each process, the score in the *y*-axis highlights its level of significance as -ln(*q*-value). The size of each bubble is proportional to the amount of DEGs implicated in the process compared to the number of genes known to be associated with the process itself.

To study the main sub-networks, we inspected the overrepresented biological processes in which the DEGs highlighted in Figure 1 are involved. We identified 205 overrepresented biological processes, and we focused on the 22 processes that are implicated in our study. As it is possible to observe in Figure 3, these processes are related to cell cycle, brain organization, immune response and oxidative stress. As expected, most of the processes are related to the immune response.

**Figure 4:**
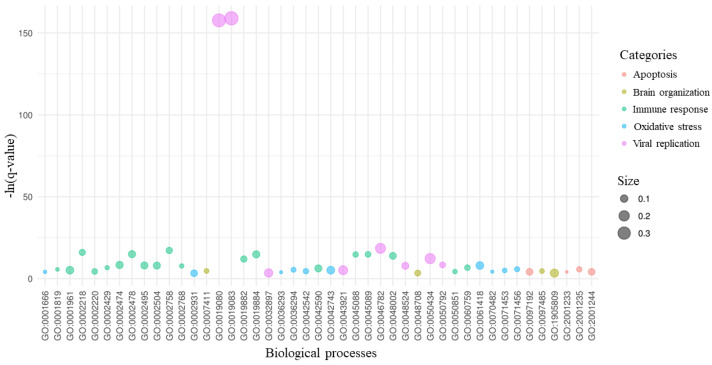
Biological processes of the key DEGs. For the 21 DEGs with the highest betweenness centrality and their 244 interactors, we provided the gene ontology enrichment of their biological processes. Among all the statistical significant processes, we kept the 45 processes related to apoptosis, brain organization, immune response, oxidative stress and viral replication, as shown in the legend. On the *x*-axis, we listed the gene ontology IDs of all the 45 processes. For each process, the score in the *y*-axis highlights its level of significance as -ln(*q*-value). The size of each bubble is proportional to the amount of DEGs implicated in the process compared to the number of genes that are known to be associated with the process itself.

Again, we made the same inspection for DEGs of the AD against AD+COVID-19 comparison in Figure 1. Among the 499 overrepresented biological processes, 45 processes are in compliance with our study. Figure 4 shows the processes related to apoptosis, brain organization, immune response, oxidative stress and viral activity. It is interesting that “viral gene expression” (GO:0019080) and “viral transcription” (GO:0019083) have a very high score compared to the other gene ontologies, and they are the processes that included the most of DEGs.

## 3. Discussion

The COVID-19 pandemic is killing millions of people worldwide. The death rate of COVID-19 is higher in the elderly and in individuals with comorbidity. Additionally, COVID-19 seems to trigger or, at least, accelerate the neurological disorders in people infected by SARS-CoV-2. Indeed, viral infections are known to impair the structure of the central or peripheral nervous system, hindering its functionality. The human coronaviruses HCoV-229E, HCoV-OC43, HCoV-NL63, HCoV-HKU1, SARS-CoV and MERS-CoV are neurotropic viruses that are able to trigger neurotoxicity, causing nervous system damage through demyelination, inflammatory state and hypoxia injury [14]. The consequence of SARS-CoV infections revealed neurons ischemia and nerve demyelination, polyneuropathy, encephalitis and stroke [15,16]. In spite of SARS-CoV, neurotoxicity induced by MERS-CoV triggers insanity, disturbance of consciousness, paralysis, Guillain–Barré syndrome or neuropathy not directly linked to respiratory distress [17,18]. SARS-CoV-2 seems to infect the olfactory nerves; therefore, the frontal cortex could be the access route for the virus to the brain [19]. Among the people with COVID-19, patients already suffering from neurological disorders, such as dementia and especially AD, tend to have a higher rate of infection and death. Nevertheless, it is not clear what mechanisms exacerbate the disease of patients with dementia [12].

In this study, we retrieved by GEO repository a cohort of control individuals, a cohort of patients who died due to AD and a cohort of patients with AD who died after SARS-CoV-2 diagnosis. The mean age of the control cohort is 78.4 years old, the COVID-19 cohort’s is 79, the AD cohort’s is 89.1 and the AD+COVID-19 cohort’s is 84.8; thus, they are very close. Other comorbidities were highlighted in the patients, but AD is the only common disease observed. Thus, we inspected the transcriptomic profile obtained by the frontal cortex of the individuals in the four cohorts. Indeed, the frontal cortex, which is involved in memory, planning and reasoning, is also compromised in advanced AD [11].

We focused our attention on the DEGs obtained by first comparing the control cohort against the COVID-19 cohort and then the AD cohort against the AD+COVID-19 cohort. Once the transcriptomic profile was reconstructed, we analyzed the interactome in which the DEGs are involved. Indeed, the alteration of these genes can cause the destruction of whole interactomes. In the first comparison, for the control against COVID-19, we recognized *F2*, *CDC25A*, *CDC25C*, *PRKACG*, *NFATC1*, *PPARG* and *MAPK3* as being upregulated, as shown in Table 1. *F2* encodes for the coagulator factor II, the prothrombin protein, and its upregulation fits with several clinicopathological reports. Indeed, even if the mechanism is not clearly elucidated, the association between COVID-19 and coagulopathy or thrombosis is known as the platelet drop and thrombin formation [20]. *CDC25A* and *CDC25C* are phosphatases belonging to the Cell Division Cycle (CDC) 25 family. Both proteins regulate the cell cycle and are upregulated. Particularly, *CDC25A* is required by the cell to switch from the G1 to S phase, while *CDC25C* is necessary to trigger the mitosis. Viruses of influenza are known to modulate the expression of the CDC25 family cell-cycle proteins, increasing their fitness [21]. Additionally, Lv et al. observed that the porcine hemagglutinating encephalomyelitis virus, a neurotropic virus of the Coronariviridae family, modulates *CDC42.* The modulation culminates in the rearrangement of the actin cytoskeleton; thus, the virus can survive easily [22]. *NFATC1*, the Nuclear Factor of Activated T Cells 1 (NFAT) protein, is a transcription factor of the NFAT family. In particular, *NFATC1* is the cytosolic component of the complex that enters into the nucleus after the stimulation of the T-cell receptors. While NFATs are involved in the development of several diseases and disorders, they are known as regulators of the immune response mediated by T cells in response to cytokines. The upregulation of the *NFATC1* underlines the presence of the virus in the brain. Indeed, following coronavirus infection, the immune response, i.e., NFAT complex, is activated by virus envelope protein detection by which the virus virulence also depends [23]. The presence of the SARS-CoV-2 in the brain can also be hypothesized by the upregulation of *PPARG*. *PPARG* encodes for the Peroxisome Proliferator Activated Receptor (PPAR) Gamma, a member of the PPAR family. Cao et al. observed the immune response in mice that, in line with our analysis, shows a quick increase of PPAR-γ expression [24]. 

*PRKACG* is a catalytic subunit of the Protein Kinase A (PKA). Interestingly, the role of PKA was investigated in compliance with viral replication. Indeed, PKA could translocate the V-ATPASE into lysosome, allowing the release of viral RNA [25].

*MAPK3* is a Mitogen-Activated Protein Kinase (MAPK) involved in MAPK pathway. *MAPK3* encodes for ERK, and its role in activation, proliferation, differentiation, cell cycle progression and apoptosis is very well known [26]. The role of the ERK in MAPK pathways has already been studied with other viruses that, through overactivation of this protein, as in our study, improve their viral replication [27]. 

As expected, in patients without dementia, we simply observed the effects of the SARS-CoV-2, as summarized by the Figure 3. Indeed, the main biological processes in which are involved the central DEGs of the comparison and their interactors (Figure 1) are related to immune response, cell cycle, oxidative stress and brain organization.

Conversely, Table 2 shows the 21 central genes in the comparison of the AD cohort against the AD+COVID-19 cohort (Table 2). In detail, *RPS27A*, *UBA52*, *NCOR1*, *MAPK3*, *NCOR2*, *SNW1*, *RAC1*, *NCL*, *PTPN11*, *POL2RA* and *ARRB1* are upregulated, while *ESR1*, *KRAS*, *PTGES3*, *RB1*, *SEC13*, *MAPK14*, *TBL1XR1*, *SRSF1*, *DUT* and *NCBP1* are downregulated. 

*RPS27A* and *UBA52* are two of the four human ubiquitins, and they encode for a fusion ribosomal protein. Several neurodegenerative disorders, such as AD, have ubiquitins implicated to reduce neurotoxicity binding specific aggregates [28]. The ubiquitins target cellular protein to degrade them by the proteosome, and they are essential to the cellular steady state [29]. Herein, they are the coding proteins with the highest betweenness centrality (0.20 and 0.15, respectively), they have the highest degrees (103 and 95) and they both are upregulated. The organism reacts to harmful proteins accumulation induced by stress through the ubiquitin protein system; thus, with an increase in the expression of the ubiquitins. The ubiquitin proteosome dysregulation is a characteristic of neuro-infectious diseases through the regulation of oxidative and inflammatory stress, as well as immunity response. Particularly, in neurons and glial cells, the immunoproteasome is significantly upregulated in patients with AD, and it is induced by inflammatory cytokine and oxidative stress [30]. Chen et al. showed that ubiquitin immunoreactivity is caused by hypoxic injury, along with mitochondrial dysfunction and an increase in reactive oxygen species, phopho-tau and beta amyloid [31]. Thus, we can hypothesize that the immune response is exacerbated in patients with AD+COVID-19 because of the upregulation of ubiquitins in a condition in which the immunoproteasome is already upregulated for the presence of the AD itself.

Our analysis also shows the downregulation of *TBL1XR1* and *ESR1*, as well as the upregulation of *NCOR1* and *NCOR2*. *TBL1XR1* encodes for the F-box-like/WD repeat-containing protein. The WD repeat-containing proteins are nuclear receptors involved in the differentiation program of the cell, and they are needed by several transcription factors. Its downregulation highlights a reduced activity that matches with the upregulation of *NCOR1* and *NCOR2*. Indeed, *NCOR1* and *NCOR2* encode respectively for the nuclear receptor co-repressors 1 and 2. They act as coregulatory transcriptional factors of TBLX family and repress several classes of transcription factors recruiting histone deacetylases. *TBL1XR1* could also be a component of N-COR corepressor complex from which the proteosome complex is recruited [32]. However, not all the specific targets of NCOR machinery have yet been identified. Ogawa et al. identified, in the protein encoded by *JUN*, a subunit of AP-1 transcription factor, a recruiter of NCOR implicated in inflammatory signaling [33]. In our analysis, *JUN* was not deregulated, but, on the other hand, *JUND* was upregulated. Interestingly, *JUND* is a gene of the JUN family and a functional component of the complex AP-1, so that its deregulation could lead to the same biological effect. Just as *TBL1XR1*, *SEC13* contains a WD repeat-containing protein and it is a component of the nuclear complex. Moreira et al. inspected a mouse model with a low level of Sec13 protein that altered the level of immune factors hindering a functional inflammatory response [34]. Moreover, Sec13 interacts with presenilin-1, a protein that, when mutated, triggers the neurotoxicity in AD through the alteration of the protein-degradation pathway [35].

*ARRB1* encodes for the Arrestin Beta 1. The Arrestin family is quite involved in the regulation of the nervous system through the modulation of hormones, sensory signals and neurotransmitters. Liu et al, in an in vivo study of mice, observed the role of the Arrestin Beta 1 in AD. The upregulation of the protein correlates with the severity of AD, because it modulated the γ-secretase complex, and consequently with the beta-amyloid production increasing neurotoxicity [36].

*ESR1* encodes a nuclear receptor called the estrogen receptor alpha. It is activated by estrogen, and it has a key role in central-nervous-system maintenance. Indeed, estrogen has already been associated in several studies with neuroprotective effects, and it can mediate the toxicity of beta amyloid, oxidative stress and inflammation [37]. Interestingly, Kelly et al. observed, through the Western blot technique, a reduction in the level of the estrogen receptor alpha in frontal cortex that could be associated with the progression of AD [38]. In line with this study, our AD+COVID-19 cohort shows the downregulation of the *ESR1* receptor; thus, neurotoxicity cannot be reduced.

*PTGES3* encodes for the prostaglandin E synthase 3, a protein that synthesizes prostaglandin E2 from prostaglandin endoperoxide H2, and it is downregulated in AD+COVID-19. The role of the prostaglandins had already been associated with the AD pathogenesis. Indeed, prostaglandins are downstream effectors of Cox proteins, and their alteration can lead to neurotoxicity, neuroinflammation and apoptosis [39]. *PTGES3* is a co-chaperone of heat shock proteins 90 involved in the mediation of neuroinflammatory response, and it is already known that viruses, such as SARS-CoV-2, use heat shock proteins for replication [40]. 

*MAPK14* is an MAPK that encodes for p38, and it is usually activated in response to different types of cellular stresses and leads to apoptosis. Curiously, the downregulation of *MAPK14* in our study could represent a protective mechanism operated by SARS-CoV-2 to avoid apoptosis, as observed in other viral infections [41].

*KRAS* and *RAC1* encode for GTPase and are respectively downregulated and upregulated in AD+COVID-19. *KRAS* plays a role as molecular switch of the MAPK pathway, and it is essential for brain status. Indeed, *KRAS* is a member of the RAS family that is involved in neuronal survival, regeneration, differentiation or apoptosis, as well as synaptic connectivity or cytoskeletal integrity [42]. The association of *RAC1* with AD is already known. Even if the *RAC1* results are contradictory, it seems that its expression level could be related to the grade of the AD. Indeed, Borin et al. demonstrated that *RAC1* overexpression, as in our study, increases fragments of pathogenic beta-amyloid that culminates in the hyper-phosphorylation of tau protein, thus enhancing the neurotoxicity [43]. *RAC1*—along with *SEC13*, *ARRB1* and *ESR1*, which we have already described—highlights the impairment of the beta-amyloid clearance in AD patients in which the neurotoxicity mediated by the beta-amyloid plaques is already high. Additionally, Matsui et al. showed that extensive neuronal death in AD leads to a decrease in the expression of *RAC1* [43]. Notably, the consequence of *RAC1* overexpression finds a match with the overexpression of *PTPN11* that encodes for the Protein Tyrosin Phosphatase Non-Receptor Type 11. Indeed, Kim et al. speculated the direct interaction between the hyperphosphorylated tau and the phosphatase encoded by *PTPN11* and its role in neurodegeneration in AD patients. In particular, *PTPN11* overexpression in brain seems to correlate with the severity of AD [44]. Thus, the parallel overexpression of *RAC1* and *PTPN11* in AD patients with COVID-19 could be important for the enhancing of neurotoxicity.

*RB1* encodes for the retinoblastoma protein involved in the negative regulation of the cell cycle. It was downregulated in our analysis, and the knockout of retinoblastoma was inspected in a mice study of MacPherson et al. As expected, the loss of the retinoblastoma alters the cell-cycle activity and apoptosis in the nervous system; an increase in the hypoxia-inducible genes is also observed [45].

*NCL* encodes for nucleolin, a nucleolar protein involved in cell cycle and DNA damage. In a previous study conducted by Jang et al. about Parkinson’s disease, *NCL* overexpression was associated to neurotoxicity induced by oxidative stress. *NCL* is overexpressed in AD+COVID-19 cohort, but Jang et al. associated its protective role with the overexpression of heat shock protein 70 [46]. In our results, we observed the downregulation of the heat shock protein 70 *HSPA13* and *HSPA1B*, so we can hypothesize that nucleolin cannot protect the cells from the neurotoxicity induced by oxidative stress.

*POL2RA* encodes for the subunit A of the RNA polymerase II, while *SNW1* enhances the transcription as a coactivator of polymerase II. In our results, we observed an increase in the expression of both *POL2RA* and *SNW1* transcripts. Interestingly, the role of polymerase II had already been associated with AD. Specifically, the hyperphosphorylation of the RNA polymerase II promotes the neurodegeneration, hindering the synthesis of mRNA that induces brain atrophy [47]. This observation could be strengthened by the downregulation of *NCBP1*. This gene is the Subunit 1 of the Nuclear Cap Binding Protein, and it is necessary for the export of mRNA [48]. Moreover, the Serine- and Arginine-Rich Splicing Factor 1 is encoded by *SRSF1*, which is downregulated in AD+COVID-19 cohort. *SRSF1* activity promotes the splicing and has been observed to be deregulated in AD patients consequently to hypoxia associated with neurodegeneration. Specifically, *SRSF1* upregulation seems to promote the inclusion of exon 10 of Tau protein, but SRSF1 downregulation changes the ratio between three repeats and four repeats of the exon 10 [49].

The Deoxyuridine Triphosphatase protein is encoded by *DUT*. Williams et al. observed the role of this protein related to apoptosis. Interestingly, it could serve as a pro-apoptotic second messenger under the presence of reactive oxygen species [49]. Thus, the downregulation of *DUT* confirms the hindering of the apoptosis as a mechanism of the virus that tries to replicate itself as much as possible [50].

**Figure 5:**
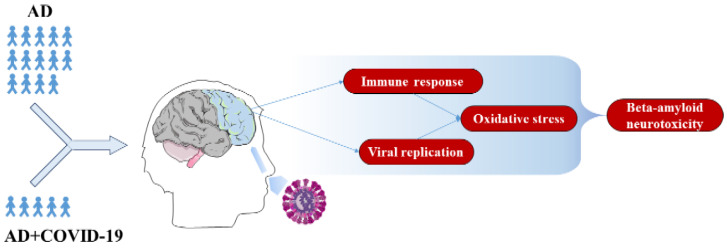
Summary of the effects of COVID-19 in AD patients. The olfactory epithelium is the possible access route of SARS-CoV-2 in the human brain; thus, the frontal cortex area should be the first brain region to be damaged from the invasion. The comparative analysis of 14 individuals who died with AD against 5 patients who died from COVID-19 (AD+COVID-19) revealed a reduction in apoptosis that leads to a prolonged viral replication. Along with immune response activation, SARS-CoV-2 increases the oxidative stress that culminates in enhanced beta-amyloid neurotoxicity.

In addition, the key DEGs here described directly modulate the 244 DEGs shown in Figure 2. To support the evidence found in the literature about the 21 key DEGs here described, we also inspected the overrepresented gene ontologies of the biological process for the 244 DEGs. It is interesting that we found the overrepresentation of 45 biological processes that we clustered in the five categories, namely apoptosis, brain organization, immune response, oxidative stress and viral replication (Figure 4). Figure 5 summarizes the conclusion of our analysis. Indeed, in line with our study, the infection from SARS-CoV-2 significantly increases inflammation in response to viral entry. Consequently, oxidative stress could be induced by hypoxia condition, and the physiological apoptosis should be altered [51]. Taken together, all of these considerations suggest an increase in beta-amyloid neurotoxicity and a worsening of neurodegeneration.

## 4. Materials and Methods

### 4.1. Cohort Selection

We retrieved from GEO repository the datasets PRJNA690013 [52] and PRJNA232669 [53] that collect transcriptional data from frontal cortex. 

The dataset PRJNA690013 collects 16 runs of individual sequences, using the Illumina instrument NextSeq 500. The dataset consists of 7 controls and 9 patients who died of COVID-19. Among the control individuals, 6 of them suffered from dementia. In addition, 6 of the patients with COVID-19 suffered from dementia. We did not download the control run SRR13367152 and the COVID-19 run SRR13367158, because they suffered only from vascular dementia. The remaining samples had dementia associated with AD. Finally, this cohort was composed of 5 males and 9 females. Among them, cardiovascular disease, diabetes, obesity, pulmonary disorders, vascular dementia and/or cancer were comorbidities observed for 8 out of 10 patients. 

To enlarge the cohort of control and AD patients, we used the dataset PRJNA232669 that collects 17 runs sequenced through Illumina instrument HiSeq 2500. The dataset is made of 9 patients who suffered from advanced AD and 8 control individuals. No information about sex or other comorbidities is provided for the dataset.

The aim of our work was to establish why AD patients with COVID-19 are likely to be infected with SARS-CoV-2 and, eventually, worsen their condition, so we first compared the cohorts of control against COVID-19 and then AD against AD+COVID-19 individuals. The control cohort is made of runs SRR2422918, SRR2422919, SRR2422920, SRR2422921, SRR2422922, SRR2422923, SRR2422924, SRR2422925 (control individual of PRJNA232669) and SRR13367151 (control individual without dementia of PRJNA690013). The runs SRR13367159, SRR13367161 and SRR13367162 (COVID-19 patients without dementia of PRJNA690013) build the COVID-19 cohort. The AD cohort consists of runs SRR2422926, SRR2422927, SRR2422928, SRR2422929, SRR2422930, SRR2422931, SRR2422932, SRR2422933, SRR2422934 (AD patients of PRJNA232669) SRR13367150, SRR13367153, SRR13367154, SRR13367155 and SRR13367156 (AD patients of PRJNA690013). The AD+COVID-19 cohort is made of runs SRR13367157, SRR13367160, SRR13367163, SRR13367164 and SRR13367165 (COVID-19 patients with AD of PRJNA690013). In summary, the control, COVID-19, AD and AD+COVID-19 cohorts have a mean age of 78.4, 79, 89.1 and 84.8 years old, respectively.

### 4.2. Bioinformatics Analysis

The control, COVID-19, AD and AD+COVID-19 cohorts were downloaded by using fastq-dump version 2.8.2 [54], and the quality check was confirmed with fastqc 0.11.4 (Babraham Institute, Cambridge, UK). The bases with a low quality score, along with the adapters, were dropped with Trimmomatic 0.38 (Usadel Lab, Aachen, Germany) [55]. Then the reads were aligned against the GRCh38 human reference genome, using the Spliced Transcripts Alignment to a Reference (STAR) RNA-seq aligner 2.7.3a (New York, NY, USA) [56], and the transcripts count was obtained with the python package htseq-count 0.6.1p1 (European Molecular Biology Laboratory (EMBL), Heidelberg, Germany) [57]. We extracted from the full list of transcripts identified as protein coding by using the biomaRt 2.40.5 [58] library in R 3.6.3 (R Core Team). Finally, the analysis of DEGs was performed with DESeq2 library [59]. Two different lists of DEGs were obtained from the comparisons. The first DEGs concern the control cohort against the COVID-19 cohort, whereas the second DEGs are related to the AD cohort against the AD+COVID-19 cohort. Since the focus of our work was the observation of all the DEGs that can alter the interactome stability, we did not set any fold-change cutoff. In spite of the fold change, we used the post hoc Benjamini–Hochberg correction to filter out the false positive DEGs with a *q*-value higher than 0.05. We also used the libraries stringr and dplyr to handle the data structures. The networks of DEGs were analyzed with the online web page of STRING [60,61]. To obtain the highest quality of the networks, we set the highest confidence level (0.900) by using “Experiments” and “Database” as sources. The networks were then exported, customized and inspected with Cytoscape 3.8.2 [62].

## 5. Conclusions

The COVID-19 pandemic induced by the SARS-CoV-2 especially worsens the condition of those suffered from chronic degenerative diseases, such as AD. In our work, the transcriptomic and interactomic analysis revealed the impairment of processes that are already known to be associated with AD condition, such as immune response, oxidative stress and apoptosis. Herein, we speculated that SARS-CoV-2 can worsen the behavior of the frontal cortex in patients with AD, increasing the neurotoxicity. Our hypothesis is strengthened by the alteration of DEGs involved in apoptosis, brain organization, immune response, oxidative stress and viral replication, as highlighted by the gene ontologies of the biological process. In fact, in response to viral replication, immune response and oxidative stress may enhance and consequentially increase the beta-amyloid neurotoxicity in the brain.

## Figures and Tables

**Table 1:** DEGs with betweenness centrality outer than 95% after z-score normalization.

Gene	Control	COVID-19	Fold Change	Betweenness Centrality	Degree
MAPK3	365.56	695.22	0.93	0.51	15
PRKACG	0.06	20.49	6.69	0.44	10
CDC25C	2.79	43.05	3.80	0.34	3
NFATC1	33.21	166.23	2.32	0.29	6
CDC25A	7.03	61.62	3.16	0.25	4
F2	1.07	33.31	5.13	0.19	9
PPARG	42.25	128.56	1.60	0.19	3

Note: The fold change was computed as log_2_(COVID-19/control). DEGs are sorted by betweenness centrality parameter. All the values are rounded to the second decimal digit.

**Table 2:** DEGs with betweenness centrality outer than 95% after z-score normalization.

Gene	AD	AD+COVID-19	Fold Change	Betweenness Centrality	Degree
RPS27A	911.74	1780.96	0.97	0.20	103
UBA52	677.56	1204.86	0.82	0.15	95
NCOR1	2542.38	3646.46	0.52	0.13	18
MAPK3	444.68	763.02	0.78	0.13	26
ESR1	74.27	19.81	−1.84	0.10	23
KRAS	673.02	411.00	−0.72	0.09	26
PTGES3	1154.09	676.38	−0.75	0.08	16
NCOR2	1127.04	2296.71	1.03	0.08	14
RB1	600.81	340.79	−0.81	0.07	16
SNW1	269.40	404.53	0.56	0.06	17
SEC13	228.79	131.39	−0.69	0.06	16
MAPK14	485.07	267.52	−0.84	0.06	18
TBL1XR1	2220.09	1191.37	−0.90	0.06	8
RAC1	948.07	1678.89	0.83	0.05	22
NCL	1784.81	6608.74	1.89	0.05	17
SRSF1	506.54	284.79	−0.78	0.05	19
PTPN11	2778.66	5429.21	0.97	0.04	16
DUT	237.24	152.47	−0.67	0.04	9
NCBP1	600.36	409.11	−0.58	0.04	14
POLR2A	1164.20	1595.16	0.45	0.04	22
ARRB1	1183.63	1826.55	0.62	0.04	10

Note: The fold change was computed as log_2_(AD+COVID-19/AD). DEGs are sorted by betweenness centrality parameter. All the values are rounded to the second decimal digit.

## Data Availability

The data presented in this study are openly available in the NCBI Sequence Read Archive at BioProject, under accession numbers PRJNA690013 and PRJNA232669.

## References

[1] World Health Organization. https://covid19.who.int/.

[2] Tay M.Z., Poh C.M., Renia L., MacAry P.A., Ng L.F.P. (2020). The trinity of COVID-19: Immunity, inflammation and intervention. Nat. Rev. Immunol..

[3] Lopez-Leon S., Wegman-Ostrosky T., Perelman C., Sepulveda R., Rebolledo P.A., Cuapio A., Villapol S. (2021). More than 50 long-term effects of COVID-19: A systematic review and meta-analysis. Sci. Rep..

[4] Thakur B., Dubey P., Benitez J., Torres J.P., Reddy S., Shokar N., Aung K., Mukherjee D., Dwivedi A.K. (2021). A systematic review and meta-analysis of geographic differences in comorbidities and associated severity and mortality among individuals with COVID-19. Sci. Rep..

[5] World Health Organization. https://www.who.int/news-room/fact-sheets/detail/dementia.

[6] Jeremic D., Jimenez-Diaz L., Navarro-Lopez J.D. (2021). Past, present and future of therapeutic strategies against amyloid-beta peptides in Alzheimer’s disease a systematic review. Ageing Res. Rev..

[7] Ferini-Strambi L., Salsone M. (2021). COVID-19 and neurological disorders: Are neurodegenerative or neuroimmunological diseases more vulnerable?. J. Neurol..

[8] Rahman M.A., Islam K., Rahman S., Alamin M. (2021). Neurobiochemical Cross-talk Between COVID-19 and Alzheimer’s Disease. Mol. Neurobiol..

[9] Ding Q., Shults N.V., Gychka S.G., Harris B.T., Suzuki Y.J. (2021). Protein Expression of Angiotensin-Converting Enzyme 2 (ACE2) is Upregulated in Brains with Alzheimer’s Disease. Int. J. Mol. Sci..

[10] Politi L.S., Salsano E., Grimaldi M. (2020). Magnetic Resonance Imaging Alteration of the Brain in a Patient With Coronavirus Disease 2019 (COVID-19) and Anosmia. JAMA Neurol..

[11] O’Reilly R.C. (2010). The What and How of prefrontal cortical organization. Trends Neurosci..

[12] Bianchetti A., Rozzini R., Guerini F., Boffelli S., Ranieri P., Minelli G., Bianchetti L., Trabucchi M. (2020). Clinical Presentation of COVID-19 in Dementia Patients. J. Nutr. Health Aging.

[13] Barrett T., Wilhite S.E., Ledoux P., Evangelista C., Kim I.F., Tomashevsky M., Marshall K.A., Phillippy K.H., Sherman P.M., Holko M. (2013). NCBI GEO: Archive for functional genomics data sets--update. Nucleic Acids Res..

[14] Wu Y., Xu X., Chen Z., Duan J., Hashimoto K., Yang L., Liu C., Yang C. (2020). Nervous system involvement after infection with COVID-19 and other coronaviruses. Brain Behav. Immun..

[15] Tsai L.K., Hsieh S.T., Chang Y.C. (2005). Neurological manifestations in severe acute respiratory syndrome. Acta Neurol. Taiwanica.

[16] Gu J., Gong E., Zhang B., Zheng J., Gao Z., Zhong Y., Zou W., Zhan J., Wang S., Xie Z. (2005). Multiple organ infection and the pathogenesis of SARS. J. Exp. Med..

[17] Kim J.E., Heo J.H., Kim H.O., Song S.H., Park S.S., Park T.H., Ahn J.Y., Kim M.K., Choi J.P. (2017). Neurological Complications during Treatment of Middle East Respiratory Syndrome. J. Clin. Neurol..

[18] Saad M., Omrani A.S., Baig K., Bahloul A., Elzein F., Matin M.A., Selim M.A., Al Mutairi M., Al Nakhli D., Al Aidaroos A.Y. (2014). Clinical aspects and outcomes of 70 patients with Middle East respiratory syndrome coronavirus infection: A single-center experience in Saudi Arabia. Int. J. Infect. Dis..

[19] Meinhardt J., Radke J., Dittmayer C., Franz J., Thomas C., Mothes R., Laue M., Schneider J., Brunink S., Greuel S. (2021). Olfactory transmucosal SARS-CoV-2 invasion as a port of central nervous system entry in individuals with COVID-19. Nat. Neurosci..

[20] Kipshidze N., Dangas G., White C.J., Kipshidze N., Siddiqui F., Lattimer C.R., Carter C.A., Fareed J. (2020). Viral Coagulopathy in Patients With COVID-19: Treatment and Care. Clin. Appl. Thromb. Hemost..

[21] Perwitasari O., Torrecilhas A.C., Yan X., Johnson S., White C., Tompkins S.M., Tripp R.A. (2013). Targeting cell division cycle 25 homolog B to regulate influenza virus replication. J. Virol..

[22] Lv X., Li Z., Guan J., Hu S., Zhang J., Lan Y., Zhao K., Lu H., Song D., He H. (2019). Porcine Hemagglutinating Encephalomyelitis Virus Activation of the Integrin alpha5beta1-FAK-Cofilin Pathway Causes Cytoskeletal Rearrangement To Promote Its Invasion of N2a Cells. J. Virol..

[23] DeDiego M.L., Nieto-Torres J.L., Jimenez-Guardeno J.M., Regla-Nava J.A., Castano-Rodriguez C., Fernandez-Delgado R., Usera F., Enjuanes L. (2014). Coronavirus virulence genes with main focus on SARS-CoV envelope gene. Virus Res..

[24] Cao X., Tian Y., Nguyen V., Zhang Y., Gao C., Yin R., Carver W., Fan D., Albrecht H., Cui T. (2021). Spike protein of SARS-CoV-2 activates macrophages and contributes to induction of acute lung inflammation in male mice. FASEB J..

[25] Aslam M., Ladilov Y. (2020). Targeting the sAC-Dependent cAMP Pool to Prevent SARS-Cov-2 Infection. Cells.

[26] Peyssonnaux C., Eychene A. (2001). The Raf/MEK/ERK pathway: New concepts of activation. Biol. Cell.

[27] Ghasemnejad-Berenji M., Pashapour S. (2021). SARS-CoV-2 and the Possible Role of Raf/MEK/ERK Pathway in Viral Survival: Is This a Potential Therapeutic Strategy for COVID-19?. Pharmacology.

[28] Boland B., Yu W.H., Corti O., Mollereau B., Henriques A., Bezard E., Pastores G.M., Rubinsztein D.C., Nixon R.A., Duchen M.R. (2018). Promoting the clearance of neurotoxic proteins in neurodegenerative disorders of ageing. Nat. Rev. Drug Discov..

[29] Kimura Y., Tanaka K. (2010). Regulatory mechanisms involved in the control of ubiquitin homeostasis. J. Biochem..

[30] Limanaqi F., Biagioni F., Gaglione A., Busceti C.L., Fornai F. (2019). A Sentinel in the Crosstalk Between the Nervous and Immune System: The (Immuno)-Proteasome. Front. Immunol..

[31] Chen G.J., Xu J., Lahousse S.A., Caggiano N.L., de la Monte S.M. (2003). Transient hypoxia causes Alzheimer-type molecular and biochemical abnormalities in cortical neurons: Potential strategies for neuroprotection. JAD.

[32] Perissi V., Aggarwal A., Glass C.K., Rose D.W., Rosenfeld M.G. (2004). A corepressor/coactivator exchange complex required for transcriptional activation by nuclear receptors and other regulated transcription factors. Cell.

[33] Ogawa S., Lozach J., Jepsen K., Sawka-Verhelle D., Perissi V., Sasik R., Rose D.W., Johnson R.S., Rosenfeld M.G., Glass C.K. (2004). A nuclear receptor corepressor transcriptional checkpoint controlling activator protein 1-dependent gene networks required for macrophage activation. Proc. Natl. Acad. Sci. USA.

[34] Moreira T.G., Zhang L., Shaulov L., Harel A., Kuss S.K., Williams J., Shelton J., Somatilaka B., Seemann J., Yang J. (2015). Sec13 Regulates Expression of Specific Immune Factors Involved in Inflammation In Vivo. Sci. Rep..

[35] Nielsen A.L. (2009). The coat protein complex II, COPII, protein Sec13 directly interacts with presenilin-1. Biochem. Biophys. Res. Commun..

[36] Liu X., Zhao X., Zeng X., Bossers K., Swaab D.F., Zhao J., Pei G. (2013). beta-arrestin1 regulates gamma-secretase complex assembly and modulates amyloid-beta pathology. Cell Res..

[37] Pike C.J., Carroll J.C., Rosario E.R., Barron A.M. (2009). Protective actions of sex steroid hormones in Alzheimer’s disease. Front. Neuroendocrinol..

[38] Kelly J.F., Bienias J.L., Shah A., Meeke K.A., Schneider J.A., Soriano E., Bennett D.A. (2008). Levels of estrogen receptors alpha and beta in frontal cortex of patients with Alzheimer’s disease: Relationship to Mini-Mental State Examination scores. Curr. Alzheimer Res..

[39] Guan P.P., Wang P. (2019). Integrated communications between cyclooxygenase-2 and Alzheimer’s disease. FASEB J. Off. Publ. Fed. Am. Soc. Exp. Biol..

[40] Lubkowska A., Pluta W., Stronska A., Lalko A. (2021). Role of Heat Shock Proteins (HSP70 and HSP90) in Viral Infection. Int. J. Mol. Sci..

[41] Bello-Perez M., Sola I., Novoa B., Klionsky D.J., Falco A. (2020). Canonical and Noncanonical Autophagy as Potential Targets for COVID-19. Cells.

[42] Schoneborn H., Raudzus F., Coppey M., Neumann S., Heumann R. (2018). Perspectives of RAS and RHEB GTPase Signaling Pathways in Regenerating Brain Neurons. Int. J. Mol. Sci..

[43] Borin M., Saraceno C., Catania M., Lorenzetto E., Pontelli V., Paterlini A., Fostinelli S., Avesani A., Di Fede G., Zanusso G. (2018). Rac1 activation links tau hyperphosphorylation and Abeta dysmetabolism in Alzheimer’s disease. Acta Neuropathol. Commun..

[44] Kim Y., Liu G., Leugers C.J., Mueller J.D., Francis M.B., Hefti M.M., Schneider J.A., Lee G. (2019). Tau interacts with SHP2 in neuronal systems and in Alzheimer’s disease brains. J. Cell Sci..

[45] MacPherson D., Sage J., Crowley D., Trumpp A., Bronson R.T., Jacks T. (2003). Conditional mutation of Rb causes cell cycle defects without apoptosis in the central nervous system. Mol. Cell. Biol..

[46] Jang J., Oh H., Nam D., Seol W., Seo M.K., Park S.W., Kim H.G., Seo H., Son I., Ho D.H. (2018). Increase in anti-apoptotic molecules, nucleolin, and heat shock protein 70, against upregulated LRRK2 kinase activity. Anim. Cells Syst..

[47] Husseman J.W., Hallows J.L., Bregman D.B., Leverenz J.B., Nochlin D., Jin L.W., Vincent I. (2001). Hyperphosphorylation of RNA polymerase II and reduced neuronal RNA levels precede neurofibrillary tangles in Alzheimer disease. J. Neuropathol. Exp. Neurol..

[48] Gebhardt A., Habjan M., Benda C., Meiler A., Haas D.A., Hein M.Y., Mann A., Mann M., Habermann B., Pichlmair A. (2015). mRNA export through an additional cap-binding complex consisting of NCBP1 and NCBP3. Nat. Commun..

[49] Jakubauskiene E., Vilys L., Peciuliene I., Kanopka A. (2021). The role of hypoxia on Alzheimer’s disease-related APP and Tau mRNA formation. Gene.

[50] Best S.M. (2008). Viral subversion of apoptotic enzymes: Escape from death row. Annu. Rev. Microbiol..

[51] Alwazeer D., Liu F.F., Wu X.Y., LeBaron T.W. (2021). Combating Oxidative Stress and Inflammation in COVID-19 by Molecular Hydrogen Therapy: Mechanisms and Perspectives. Oxidative Med. Cell. Longev..

[52] Gagliardi S., Poloni E.T., Pandini C., Garofalo M., Dragoni F., Medici V., Davin A., Visona S.D., Moretti M., Sproviero D. (2021). Detection of SARS-CoV-2 genome and whole transcriptome sequencing in frontal cortex of COVID-19 patients. Brain Behav. Immun..

[53] Scheckel C., Drapeau E., Frias M.A., Park C.Y., Fak J., Zucker-Scharff I., Kou Y., Haroutunian V., Ma’ayan A., Buxbaum J.D. (2016). Regulatory consequences of neuronal ELAV-like protein binding to coding and non-coding RNAs in human brain. eLife.

[54] Fastq-dump. https://github.Com/Ncbi/Sra-Tools.

[55] Bolger A.M., Lohse M., Usadel B. (2014). Trimmomatic: A flexible trimmer for Illumina sequence data. Bioinformatics.

[56] Dobin A., Davis C.A., Schlesinger F., Drenkow J., Zaleski C., Jha S., Batut P., Chaisson M., Gingeras T.R. (2013). STAR: Ultrafast universal RNA-seq aligner. Bioinformatics.

[57] Anders S., Pyl P.T., Huber W. (2015). HTSeq—a Python framework to work with high-throughput sequencing data. Bioinformatics.

[58] Durinck S., Spellman P.T., Birney E., Huber W. (2009). Mapping identifiers for the integration of genomic datasets with the R/Bioconductor package biomaRt. Nat. Protoc..

[59] Love M.I., Huber W., Anders S. (2014). Moderated estimation of fold change and dispersion for RNA-seq data with DESeq2. Genome Biol..

[60] Szklarczyk D., Gable A.L., Lyon D., Junge A., Wyder S., Huerta-Cepas J., Simonovic M., Doncheva N.T., Morris J.H., Bork P. (2019). STRING v11: Protein-protein association networks with increased coverage, supporting functional discovery in genome-wide experimental datasets. Nucleic Acids Res..

[61] STRING. https://string-db.org/.

[62] Shannon P., Markiel A., Ozier O., Baliga N.S., Wang J.T., Ramage D., Amin N., Schwikowski B., Ideker T. (2003). Cytoscape: A software environment for integrated models of biomolecular interaction networks. Genome Res..

